# Multi-objective Informative Frequency Band Selection Based on Negentropy-induced Grey Wolf Optimizer for Fault Diagnosis of Rolling Element Bearings

**DOI:** 10.3390/s20071845

**Published:** 2020-03-26

**Authors:** Xiaohui Gu, Shaopu Yang, Yongqiang Liu, Rujiang Hao, Zechao Liu

**Affiliations:** State Key Laboratory of Mechanical Behavior and System Safety of Traffic Engineering Structures, Shijiazhuang Tiedao University, Shijiazhuang 050043, China; yangsp@stdu.edu.cn (S.Y.); liuyq@stdu.edu.cn (Y.L.); haorj@stdu.edu.cn (R.H.); cap_0219@163.com (Z.L.)

**Keywords:** bearing, fault diagnosis, multi-objective optimization, grey wolf optimizer, wavelet filter, negentropy

## Abstract

Informative frequency band (IFB) selection is a challenging task in envelope analysis for the localized fault detection of rolling element bearings. In previous studies, it was often conducted with a single indicator, such as kurtosis, etc., to guide the automatic selection. However, in some cases, it is difficult for that to fully depict and balance the fault characters from impulsiveness and cyclostationarity of the repetitive transients. To solve this problem, a novel negentropy-induced multi-objective optimized wavelet filter is proposed in this paper. The wavelet parameters are determined by a grey wolf optimizer with two independent objective functions i.e., maximizing the negentropy of squared envelope and squared envelope spectrum to capture impulsiveness and cyclostationarity, respectively. Subsequently, the average negentropy is utilized in identifying the IFB from the obtained Pareto set, which are non-dominated by other solutions to balance the impulsive and cyclostationary features and eliminate the background noise. Two cases of real vibration signals with slight bearing faults are applied in order to evaluate the performance of the proposed methodology, and the results demonstrate its effectiveness over some fast and optimal filtering methods. In addition, its stability in tracking the IFB is also tested by a case of condition monitoring data sets.

## 1. Introduction

**Figure 1:**
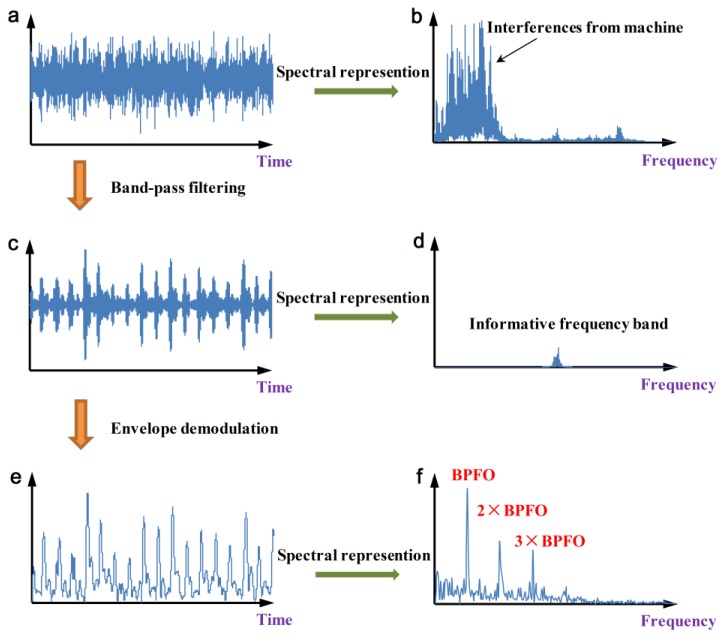
Demonstration of the envelope analysis: (**a**) original signal; (**b**) frequency spectrum; (**c**) filtered signal, (**d**) informative frequency band; (**e**) envelope and (**f**) envelope spectrum.

Rolling element bearing is a key component of rotating machines. In many cases, it operates with high speed, heavy load, and prolonged time. Thus, such localized defects as pitting, spalling, fretting, scuffing may generate on the contact surfaces. These faults will lead to abnormal vibrations, which will reduce the working accuracy and even cause catastrophic accidents [1,2]. Accordingly, the detection of the bearing failures as early as possible is an important and meaningful subject for mechanical maintenance. Vibration-based diagnostic of rolling element bearings has been interestingly investigated in recent decades [3]. As the bearing rotates, the fault-induced impulses will repetitively appear with a specific frequency that is decided by location of the defect. Generally, they are of short duration and their responses are hardly directly identified in the temporal waveform or frequency spectrum due to the background noises from machine [4]. Fortunately, these repetitive transients are excited through a resonance of the system at a much higher frequency band, which amplifies and preserves the fault information [5]. It implies that band-pass filtering the vibration signal around the resonant frequency can extract the fault features as well as eliminate the interferences. This approach is generally called the high-frequency resonance technique or envelope analysis, as illustrated in Figure 1. Hence, the most critical challenge in envelope analysis can be drawn as the informative frequency band (IFB) selection [6], which has attracted a large amount of attention in recent years [7,8,9,10,11].

Spectral kurtosis (SK) [12], as well as its fast implementation called as kurtogram [13], were viewed as a milestone for the IFB selection, in which the kurtosises of the coefficients at the output of quasi-analytic filter-banks with different central frequencies and bandwidths were employed in a frequency/frequency resolution plane for representing and detecting non-stationarities in the vibration signal. Once proposed, it has been paid a lot of attention and effort for its improvement for fault detection of rotating machines. In [14], an improved kurtogram was proposed by adopting wavelet packet transform (WPT) to replace the FIR filters to improve the accuracy of identifying the IFB and the signal-to noise ratio (SNR) of the filtered signal. After that, a new statistical index that is based on alpha-stable distribution was applied in [15] to substitute for kurtosis to characterize the non-Gaussian of fault signals. In [16], a sparsogram was further proposed while using the sparsity measurements to select the wavelet packet node from WPT. Moreover, another alternative estimator, called the Gini index, was introduced in [17] to improve the resistance of kurtosis-guided-grams to random-impulse. Recently, spectral L2/L1 norm was given in [18] to explain the SK and spectral correlation in a comprehensive way and in [19] the author compared it with kurtosis, smoothness index, and Gini index. The conclusion is that all of these popular sparsity indexes may be unfortunately affected by outliers; only some are less sensitive to outliers than others. In these above improvements of SK, the indexes are all utilized to quantitatively evaluate the band-pass filtering signal or its analytic envelope to detect the impulses can be categorized as time-domain estimators, whose biggest disadvantage is vulnerability to the outliers, as mentioned above.

Correspondingly, some frequency-domain approaches have also been proposed from another perspective to improve SK. A prime example is the protrugram [20], which selects the IFB based on the kurtosis of the envelope spectrum other than kurtosis of the time-domain demodulated signal to eliminate the negative effect of non-Gaussian outliers in the fault signals. As far as the authors know, it firstly applies the frequency-domain sparsity to characterize the fault impulses. Approximatively, an enhanced SK was put forward in [21], where the kurtosis of the power spectrum of the envelope of the filtered signal by WPT at each node was calculated to construct the kurtogram. However, the frequency-domain sparsity might result from periodic interferences, such as rotor eccentricity or gear meshing and results in fewer harmonics of fault characteristic frequencies (FCFs) in the envelope spectrum. These two aspects are all unwanted for bearing fault diagnosis. After that, measuring cyclostationarity [22] rather than the impulsiveness in IFB selection began to get more attention. In [23], kurtosis of the autocorrelation of the squared envelope (SE) rather than the direct filtered signal was utilized to generate the autogram for bearing fault diagnosis. In [24], a series of cyclostationary indicators that were based on the generalized likelihood ratio were proposed under the hypothesis of generalized Gaussian signals. In a recent work [25], a new IFB selection method that was based on the log-envelope spectrum, called log-cycligram, was given to capture the cyclostationarity separately from non-Gaussianity. Objectively, the effectiveness of the newly proposed methods [24,25] depends on the FCFs as prior knowledges. Little difference between the calculated FCF and the actual in the spectrum was required for extracting the specific repetitive transients to some extent. This flaw also existed in our previous study [26], in which the correlated kurtosis (CK) of the power spectrum was used as a novel estimator to replace kurtosis. From the above review, it can be concluded that a single time-domain, frequency-domain, or cyclostationary indicator is incomplete for bearing fault features extraction.

In recently, another interesting method that was motivated from the concepts of thermodynamics, named infogram, was proposed in [27]. As the author introduced, the negentropy of the SE of the filtered signal was employed to characterize the time-domain impulsiveness by SE infogram and negentropy of the squared envelope spectrum (SES) was employed to characterize the frequency-domain one by SES infogram. Furthermore, the average of SE infogram and SES infogram was employed to generate the average (AVE) infogram to characterize both the impulsive and cyclostationary signatures of the repetitive transients, according to Hirschman’s uncertainty principle.

The aforementioned study gave a combination of time and frequency-domain estimators for IFB selection. However, there are also limitations that are associated with it. On one hand, infogram divide the spectrum using fixed boundaries of the filter-bank like kurtogram, it might occasionally mismatch the IFB [28]. In [9], accugram was proposed based on infogram in which the health data was utilized as reference to improve its accuracy. Another effective way to deal with that is to extend the fast filtering method to optimal filtering based on evolutionary optimization [29,30,31]. In [32], the infogram was extended to Bayesian inference based optimal wavelet filter for identifying the localized defects. However, only negentropy of SE was adopted in the measurement function. It againsts the initial desire of infogram to both take impulsiveness and cyclostationarity into consideration. On the other hand, the AVE infogram was obtained by the mean of an SE infogram and SES infogram. It does not fundamentally overcome the drawback that the time-domain negentropy is not immune to the impulsive noise and the frequency-domain one is not immune to the cyclostationary noise [33,34]. However, IFB selection is easily affected by impulsive or cyclostationary noise. In addition, the impulsiveness and the cyclostationarity of repetitive transients are competitive. Hence, the sparsity of the time-domain and frequency-domain should achieve a relative balance i.e., a compromise in IFB selection. Therefore, how to balance the impulsiveness and cyclostationarity of the repetitive transients but avoid the complex interferences effectively are still worth further consideration.

A negentropy-induced multi-objective optimized wavelet filter is proposed for extracting the repetitive transients to solve the aforementioned problems. The parameters of an anti-symmetric real Laplace wavelet (ARLW) are optimized with two objective functions: maximum of the time and frequency-domain negentropy in order to characterize the impulsive and cyclostationary features, respectively. With the help of competition mechanism of multi-objective grey wolf optimizer (MOGWO) [35], some Pareto optimal solutions will be generated that are non-dominated by the others. It implies that the solution representing the most impulsive or cyclostationary components has been eliminated at iterations. Subsequently, the IFB can be easily identified by the maximum average negentropy of the Pareto set. The main contributions of the proposed method can be concluded, as follows: (1) The IFB selection is directly modeled as a multi-objective optimization problem, and it is the first attempt to solve it in bearing fault diagnosis while using the MOGWO. (2) The average negentropies of the non-dominated solutions are utilized to extract and balance the fault features. Furthermore, the maximum of average negentropies can be directly utilized to find the single optimal solution from the Pareto ones for IFB selection. It is simple enough than the knee point selection methods.

The rest of the paper is organized, as follows. In Section 2, the detail of the proposed methodology that is based on wavelet filtering and MOGWO algorithm is introduced. Next, its performances are examined and comparatively studied using several experimental signals, including two cases of incipient faults diagnosis and a case of IFB tracking in Section 3. Finally, Section 4 summarizes some conclusions.

## 2. Methodologies

### 2.1. Wavelet Transform and Anti-symmetric Real Laplace Wavelet Filter

**Figure 2:**
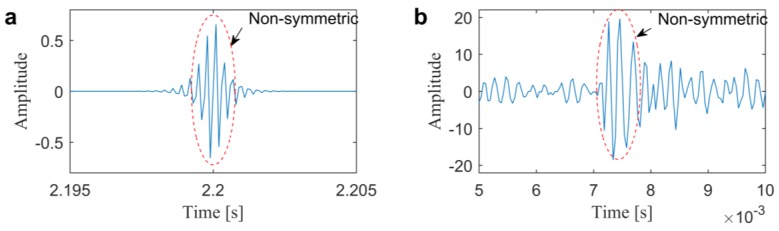
Waveform comparison of anti-symmetric real Laplace wavelet (ARLW) and the real impulse: (**a**) an artificial ARLW with γ=3400, σ=800; (**b**) an experimental bearing fault impulse.

Wavelet transform is widely used in bearing fault diagnosis and it is defined by the inner product between a wavelet ψ(t) and the analyzed signal x(t). The mathematical formula for wavelet transform can be defined as [31]:

(1) $$WT\left(a,b\right)=\int_{-\infty }^{+\infty }x\left(t\right)\times \frac{1}{\sqrt{a}}\psi^{\prime }\left(\frac{t-b}{a}\right)dt$$

where a is the scale parameter, b is the translation parameter, and $\psi^{\prime }$ is the complex conjugate of ψ. The frequency-domain equivalent of Equation (1) can be rewritten as:

(2) $$WT\left(a,b\right)=F^{-1}\left[X\left(f\right)\times \sqrt{a}\Psi \left(af\right)\right]$$

where $F^{-1}$ is the inverse Fourier transform, X(f) and Ψ(f) are the Fourier transform of x(t) and ψ(t). The ARLW is chosen in this paper that has been experimentally verified to be suitable for extracting the bearing fault features from a noisy signal, as introduced in the previous section [30]. Its temporal waveform and Fourier transform are, respectively, defined as:

(3) $$\psi \left(t\right)=e^{-\pi \sigma \left|t\right|\sin\left(2\pi \gamma t\right)}$$

(4) $$\Psi \left(f\right)=\frac{\sigma }{\pi \left[\sigma^{2}+4{\left(f-\gamma \right)}^{2}\right]i}-\frac{\sigma }{\pi \left[\sigma^{2}+4{\left(f+\gamma \right)}^{2}\right]i}$$

where γ and σ are the center frequency and bandwidth of the designed filter. It can be found that the ARLW is antisymmetric, real, exponentially damped, and has a similar morphological structure with the real fault impact responses, as shown in Figure 2. This similarity will give considerable benefits for the process of inner product, as shown in Equation (1). Afterwards, utilizing the ARLW to filter the signal x(t) through the frequency band [γ−σ/2, γ+σ/2] can be expressed as:

(5) $$WT\left(\gamma ,\sigma \right)=F^{-1}\left[X\left(f\right)\Psi^{\prime }\left(f\right)\right]$$

Subsequently, the SE and SES of the narrowband filtered signal are formed as the subsequent Equations (6) and (7):

(6) $$SE_{x}\left(\gamma ,\sigma \right)={\left|WT\left(\gamma ,\sigma \right)\right|}^{2}$$

(7) $$SES_{x}\left(\gamma ,\sigma \right)=F\left[SE_{x}\left(\gamma ,\sigma \right)\right]$$

where F denotes the Fourier transform.

In previous works, some IFB indicators were generated from Equation (6) or Equation (7), alternatively and then utilized in order to optimize the couple of γ and σ to design the optimal wavelet filter. As discussed beforehand, a single indicator is not enough to guide the correct direction in searching the IFB. Naturally, a potential and reasonable solution is to introduce the idea of multi-objective optimization, which will be detailed in the next subsection.

### 2.2. Multi-objective Optimization and Multi-objective Grey Wolf Optimizer

In fact, many real engineering problems can be modeled as a multi-objective optimization, in which there is more than one objective to be considered simultaneously, as follows:

(8) $$Maximize:\;F\left(\vec{x}\right)=f_{1}\left(\vec{x}\right),\;f_{2}\left(\vec{x}\right),\;\cdots ,\;f_{m}\left(\vec{x}\right)$$

(9) $$Subject\;to:\;\vec{x}\in \Omega$$

where $F\left(\vec{x}\right)$ consists of m objective functions, Ω is the decision space, and $\vec{x}\in \Omega$ is a decision vector. For a single-objective optimization, the solutions can be easily sorted with the help of unary objective function to select the best i.e., the optimal for decision maker. However, for a multi-objective optimization, a single optimal solution that can meet all the objectives does not usually exist. If some objectives in Equation (8) are in conflict with each other, an increment of one might cause decrement of another. In handling with multi-objective optimization, a comparison of two solutions is always done under the concept of Pareto optimality [36], which is generally defined, as follows:

**Definition** **1.** *Pareto Dominance: A vector*$\vec{x}=\left(x_{1},x_{2},\;\cdots ,x_{k}\right)$*is said to dominate another one*$\vec{y}=\left(y_{1},y_{2},\;\cdots ,y_{k}\right)$*denoted by*$\vec{x}≻\vec{y}$*, if and only if*$\forall i\in \left\{1,2,\cdots ,k\right\},\;f\left(x_{i}\right)\geq f\left(y_{i}\right)$*and*$\exists i\in \left\{1,2,\cdots ,k\right\},f\left(x_{i}\right)>f\left(y_{i}\right)$.

**Definition** **2.** *Pareto Optimality: If a solution*$\vec{x}\in X$*is said to be Pareto optimal if and only if*$∄\vec{y}\in X|F\left(\vec{y}\right)≻F\left(\vec{x}\right)$.

**Definition** **3.** *Pareto set: For a given multi-objective optimization problem, the set of all Pareto optimal solutions is called as Pareto set* $P_{s}$ *, and it is defined as:*

(10) $$P_{s}:=\left\{\vec{x},\vec{y}\in X|∄F\left(\vec{y}\right)≻F\left(\vec{x}\right)\right\}$$

**Definition** **4.** *Pareto front: For a given multi-objective optimization problem, the image of the* $P_{s}$ *in the objective space is called as Pareto front* $P_{f}$ *, and it is defined as:*

(11) $$P_{f}:=\left\{F\left(\vec{x}\right)|\vec{x}\in P_{s}\right\}$$

A meta-heuristics algorithm from the social hierarchy and hunting behavior of grey wolves named as MOGWO [35] is applied in this paper to obtain the $P_{s}$ and its corresponding $P_{f}$ of the multi-objective optimization in Equation (8). In the grey wolf optimizer (GWO) [37], the grey wolves have a strict social dominant hierarchy: the alpha (α) wolf who is the best in population, the beta (β) and delta (δ) wolves, who are the second and third best, and the rest are all classified as the omega (ω) wolves. In GWO, the hunting (optimization) is guided by α, β, and δ, the ω wolves follow these three wolves. The main phases of that include:

- Encircling prey:
  (12) $$\vec{D}=\left|\vec{C}\cdot {\vec{X}}_{p}\left(g\right)-\vec{X}\left(g\right)\right|$$
  (13) $$\vec{X}\left(g+1\right)={\vec{X}}_{p}\left(g\right)-\vec{A}\cdot \vec{D}$$
  where g denotes the current iteration, ${\vec{X}}_{p}$ is the position of prey and $\vec{X}$ is the position of the wolf. $\vec{A}$ and $\vec{C}$ are two coefficients that are defined as:
  (14) $$\vec{A}=2\vec{a}\cdot {\vec{r}}_{1}-\vec{a}$$
  (15) $$\vec{C}=2{\vec{r}}_{2}$$
  where $\vec{a}$ is linearly decreased from 2 to 0 during the iterations, and ${\vec{r}}_{1},{\vec{r}}_{2}$ are random vectors in [0, 1].
- Hunting:
  (16) $${\vec{D}}_{\alpha }=\left|{\vec{C}}_{1}\cdot {\vec{X}}_{\alpha }-\vec{X}\right|$$
  (17) $${\vec{D}}_{\beta }=\left|{\vec{C}}_{2}\cdot {\vec{X}}_{\beta }-\vec{X}\right|$$
  (18) $${\vec{D}}_{\delta }=\left|{\vec{C}}_{3}\cdot {\vec{X}}_{\delta }-\vec{X}\right|$$
  where ${\vec{X}}_{\alpha },{\vec{X}}_{\beta }$, and ${\vec{X}}_{\delta }$ indicate the best three positions attained by α, β, and δ wolves, ${\vec{D}}_{\alpha },{\vec{D}}_{\beta }$, and ${\vec{D}}_{\delta }$ represent the distances between the best three wolves and the wolf population. Subsequently, the position of the population will update under the leadership of α,β, and δ:
  (19) $${\vec{X}}_{1}={\vec{X}}_{\alpha }-{\vec{A}}_{1}\cdot {\vec{D}}_{\alpha }$$
  (20) $${\vec{X}}_{2}={\vec{X}}_{\beta }-{\vec{A}}_{2}\cdot {\vec{D}}_{\beta }$$
  (21) $${\vec{X}}_{3}={\vec{X}}_{\delta }-{\vec{A}}_{3}\cdot {\vec{D}}_{\delta }$$
  (22) $$\vec{X}\left(g+1\right)=\frac{{\vec{X}}_{1}+{\vec{X}}_{2}+{\vec{X}}_{3}}{3}$$
  where ${\vec{A}}_{1},\;{\vec{A}}_{2}$, and ${\vec{A}}_{3}$ are all generated from Equation (14) and they corresponded to α,β, and δ wolves, respectively.
- Attacking prey: When |A|>1, it indicates an exploration behavior of the search agent and otherwise indicates an exploitation one i.e., to attack the prey.
  In handling multi-objective optimization, another two operations are also introduced. Firstly, an external archive is employed to restore the non-dominated solutions with a competitive update strategy that is similar to that in [38]. Secondly, a leader selection mechanism is formulated with the help of crowding distance comparison [39] and roulette-wheel selection. The detailed introduction can be referred in [35]. The pseudo code of MOGWO algorithm is given, as below Algorithm 1:
**Algorithm 1. Multi-objective grey wolf optimizer (MOGWO).**BeginInitialize the grey wolf population $X_{i}\left(i=1,2,\cdots ,P\right)$Initialize a, A and CCalculate the objective values of each wolf, put the non-dominated solutions into the archiveSelect the best three wolves from the archive and save as α,β and δ*g* = 1**while** (*g* < maximum number of iterations)   **for** each wolf      Update the position by Equations (16)–(22)   **end for**   Update a, A and C   Calculate the objective values of each wolf, update the archive   Update α,β and δ   *g* = *g* + 1**end while**Return archive

### 2.3. Proposed Negentropy-Induced IFB Selection Method

The above MOGWO algorithm is utilized due to its fast convergence and low complexity when compared with traditional optimization techniques, such as Multi-objective particle swarm optimization (MOPSO), Multi-objective evolutionary algorithm based on decomposition (MOEA/D), etc., to properly select the wavelet parameters γ and σ of ARLW for bearing fault diagnosis. The key issue then is to design the objectives for seeking the IFB. Conventionally, the vibration responses of a normal bearing are Gaussian and stationary. When an impulse occurs, the system will deviate from its equilibrium state and generate energy fluctuations in the SE. As the bearing rotates, the energy fluctuations will be repetitive, and then harmonic frequencies will dominate in the Fourier spectrum. Based on the above considerations, the negentropy of SE and SES are proposed to characterize the impulsiveness and cyclostationarity separately, as defined as follows [27]:

(23) $$I_{SE}=\frac{1}{N}\overset{N}{\underset{n=1}{\sum }}\frac{SE_{x}\left(\gamma ,\sigma \right)}{\frac{1}{N}\sum_{n=1}^{N}SE_{x}\left(\gamma ,\sigma \right)}\ln\left[\frac{SE_{x}\left(\gamma ,\sigma \right)}{\frac{1}{N}\sum_{n=1}^{N}SE_{x}\left(\gamma ,\sigma \right)}\right]$$

(24) $$I_{SES}=\frac{1}{L}\overset{L}{\underset{\theta =1}{\sum }}\frac{SES_{x}\left(\gamma ,\sigma \right)}{\frac{1}{N}\sum_{\theta =1}^{L}SES_{x}\left(\gamma ,\sigma \right)}\ln\left[\frac{SES_{x}\left(\gamma ,\sigma \right)}{\frac{1}{L}\sum_{\theta =1}^{L}SES_{x}\left(\gamma ,\sigma \right)}\right]$$

where θ is the cyclic frequency. The average negentropy is calculated based on the Hirschman’s uncertainty principle to jointly consider these two aspects of repetitive transients:

(25) $$I_{1/2}=\left(I_{SE}+I_{SES}\right)/2$$

**Figure 3:**
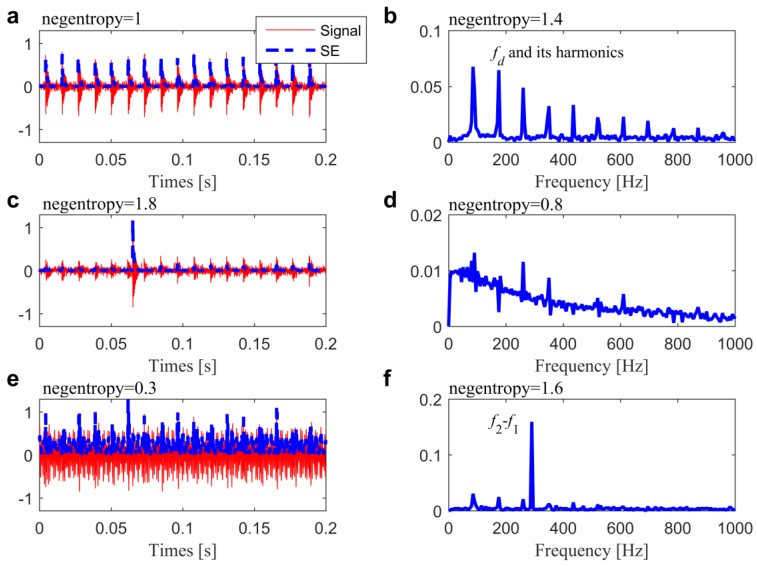
Three simulated signals together with their squared envelopes in (**a**,**c**,**e**), and the associated Fourier spectrums in (**b**,**d**,**f**).

Subsequently, the $I_{1/2}$ was employed to select the IFB directly in [27]. To illustrate its drawback in balancing the impulsiveness and cyclostationarity, negentropy of the SEs (the blue dot lines in Figure 3) and SESs (the blue solid lines in Figure 3) of three simulated filtered signals (the red thin lines in Figure 3) are analyzed. Signal 1, as plotted in Figure 3a, is generated from the following model:

(26) $$x\left(t\right)=A\left(t\right)\underset{n}{\sum }h\left(t-n/f_{d}\right)+n\left(t\right)$$

where A(t) is the impulse amplitude sampled from the uniform distribution U(0.75,0.85), n(t) is Gaussian white noise with the mean of zero and standard deviation of 0.05, and h(t) is the impulse response function, which is defined by:

(27) $$h\left(t\right)=\{\begin{array}{cc} e^{-\lambda t}\sin\left(2\pi f_{0}t\right), & t>0\\ 0, & t\leq 0 \end{array}$$

where $f_{0}=3200$ and λ=560. Signal 2 (Figure 3c) and Signal 3 (Figure 3) are also generated on the basis of Equation (26) with A(t)~U(0.25,0.35) and A(t)~U(0.45,0.55). Besides, a high-amplitude impulse noise i(t) is added in Signal 2 and two harmonic interferences c(t) are added in Signal 3, as:

(28) $$c\left(t\right)=0.2\times \sin\left(2\pi f_{1}t\right)+0.4\times \sin\left(2\pi f_{2}t\right)$$

where $f_{1}=780$ and $f_{2}=1070$. From the calculated values of SE and SES negentropy shown in Figure 3, it can be found that the maximum negentropy of the SE is attributed to the impulsive noise i(t), while the maximum negentropy of the SES is attributed to the harmonic interferences c(t). Furthermore, the biggest average negentropy indicates the Signal 2, but not the clearest impulse-train in Signal 1. Accordingly, the average negentropy as a compound index cannot evidence repetitive transients as well for the impulsiveness might completely cover up cyclostationarity and vice versa, especially when interfering contents. Therefore, it is difficult to solve this problem in the framework of single-objective optimization. Otherwise, there is a compromise in evaluating the repetitive transients in Signal 1 from SE and SES, which is consistent with the essence of Pareto optimality.

According to above analysis, two independent objectives for the IFB selection can be given, as:

(29) $$f_{1}\left(\gamma ,\sigma \right)=\frac{1}{N}\overset{N}{\underset{n=1}{\sum }}\frac{SE_{x}\left(\gamma ,\sigma \right)}{\frac{1}{N}\sum_{n=1}^{N}SE_{x}\left(\gamma ,\sigma \right)}\ln\left[\frac{SE_{x}\left(\gamma ,\sigma \right)}{\frac{1}{N}\sum_{n=1}^{N}SE_{x}\left(\gamma ,\sigma \right)}\right]$$

(30) $$f_{2}\left(\gamma ,\sigma \right)=\frac{1}{L}\overset{L}{\underset{\theta =1}{\sum }}\frac{SES_{x}\left(\gamma ,\sigma \right)}{\frac{1}{N}\sum_{\theta =1}^{L}SES_{x}\left(\gamma ,\sigma \right)}\ln\left[\frac{SES_{x}\left(\gamma ,\sigma \right)}{\frac{1}{L}\sum_{\theta =1}^{L}SES_{x}\left(\gamma ,\sigma \right)}\right]$$

**Figure 4:**
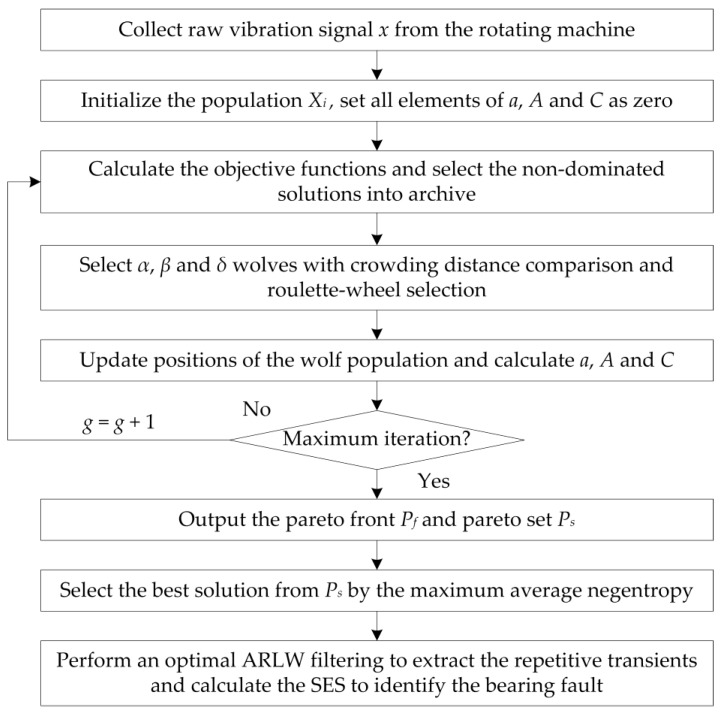
The flow diagram of the proposed method.

With the help of MOGWO, a set of Pareto optimal solutions rather than a single optimal one is obtained, but these elite candidates are selected by both the impulsiveness and cyclostationarity. Subsequently, the average negentropy of $P_{s}$ can be calculated and the IFB i.e., the single optimal solution can be determined by ranking the average values. With the help of negentropy as specific indicator and the Hirschman’s uncertainty principle, it is easy enough to pick one “best” solution out of this large set of alternatives when compared with some traditional knee point selection methods [40,41,42] by calculating the reflex angle of the points in $P_{f}$. After the selection of IFB, optimal ARLW demodulation is performed in order to detect the bearing fault due to the repetitive transients in the temporal waveform, as well as the characteristic frequencies in the Fourier spectrum, which are calculated by:

(31) $$BPFO=\frac{nF_{r}}{2}\left(1-\frac{d}{D}\cos\phi \right)$$

(32) $$BPFI=\frac{nF_{r}}{2}\left(1+\frac{d}{D}\cos\phi \right)$$

(33) $$FTF=\frac{F_{r}}{2}\left(1-\frac{d}{D}\cos\phi \right)$$

(34) $$BSF=\frac{DF_{r}}{2d}\left[1-{\left(\frac{d}{D}\cos\phi \right)}^{2}\right]$$

where $F_{r}$ is the shaft frequency, n is the number of elements, d is element diameter, D is bearing pitch diameter, and ϕ is the contact angle. Figure 4 shows the flow diagram of the proposed method. The source codes of MOGWO and the proposed method can be found in https://ww2.mathworks.cn/matlabcentral/fileexchange/55979-multi-objective-grey-wolf-optimizer-mogwo and https://ww2.mathworks.cn/matlabcentral/fileexchange/74529-negentropy-induced-mogwo.

## 3. Experimental Studies

### 3.1. Case 1: Detection of Slight Artificial Outer Race Fault in a Ball Bearing

**Figure 5:**
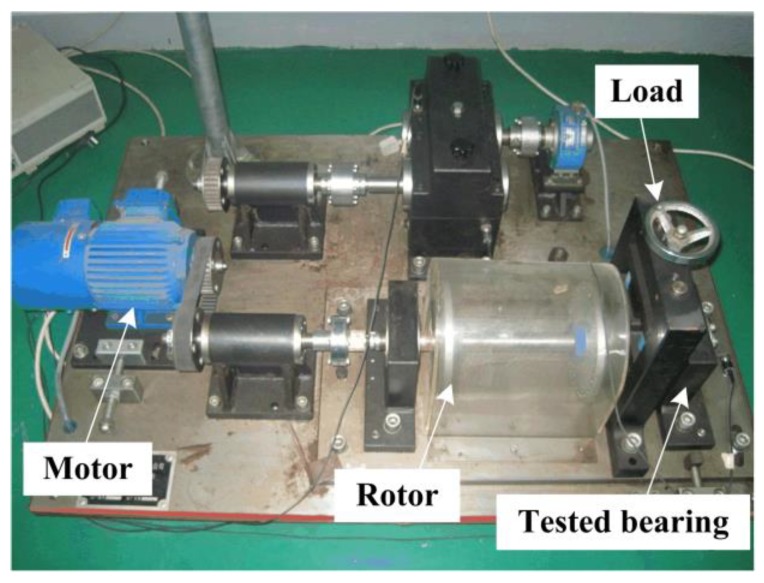
Experimental set-up for bearing fault detection in Case 1 and Case 2.

In this case, the effectiveness of the proposed method is firstly evaluated by the vibration signal that was collected by a PCB 352C33 accelerometer with the sampling frequency of 25.6 kHz from the test set-up shown in Figure 5. A single-point defect was introduced on the outer race of a NSK 6205RS bearing using electro-discharge machining with diameter of 0.2 mm to simulate the early failure in engineering. Table 1 lists the parameters of the tested bearings and manual conditions. According to Equations (31)–(34), the bearing outer race fault frequency BPFO, the inner race fault frequency BPFI, the roller spinning frequency BSF and the fundamental cage frequency FTF can be calculated as 88.2, 133.2, 58.0, and 10.0 Hz, respectively.

**Figure 6:**
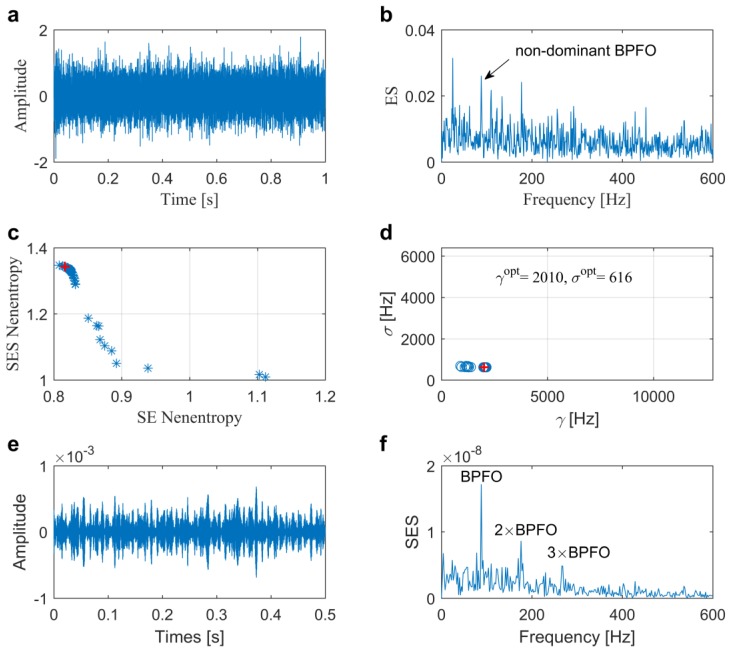
Results of the proposed method for the slight outer race fault diagnosis in Case 1: (**a**) raw signal; (**b**) envelope spectrum; (**c**) Pareto front; (**d**) distribution of the Pareto set; (**e**) filtered signal and (**f**) its associated squared envelope spectrum (SES).

Figure 6a,b plot temporal waveform and its Hilbert envelope spectrum (ES) of the original vibration signal from the tested bearing. From that, we cannot find clear repetitive transients, but only a non-dominant bearing fault characteristic frequency BPFO, which implies that further IFB selection is needed for the diagnosis. The proposed method illustrated in Figure 4 is applied to select the proper wavelet parameters. The number of population *P* and maximum generation number *G* are all set to 50. After iterations, the $P_{f}$ and $P_{s}$ are calculated, as shown in Figure 6c,d. The average negentropy values are calculated with the maximum value of 1.0797 to select the best optimal one which is marked by a red **+**. Subsequently, the optimal couple of γ and σ are identified as 2010 Hz and 616 Hz, respectively. In Figure 6e,f, the temporal waveform filtered from the ARLW and its associated SES are depicted, respectively. The results can clearly indicate the outer race fault in the bearing due to the clear repetitive transients in Figure 6e and dominated BPFO, as well as its harmonics in Figure 6f.

**Figure 7:**
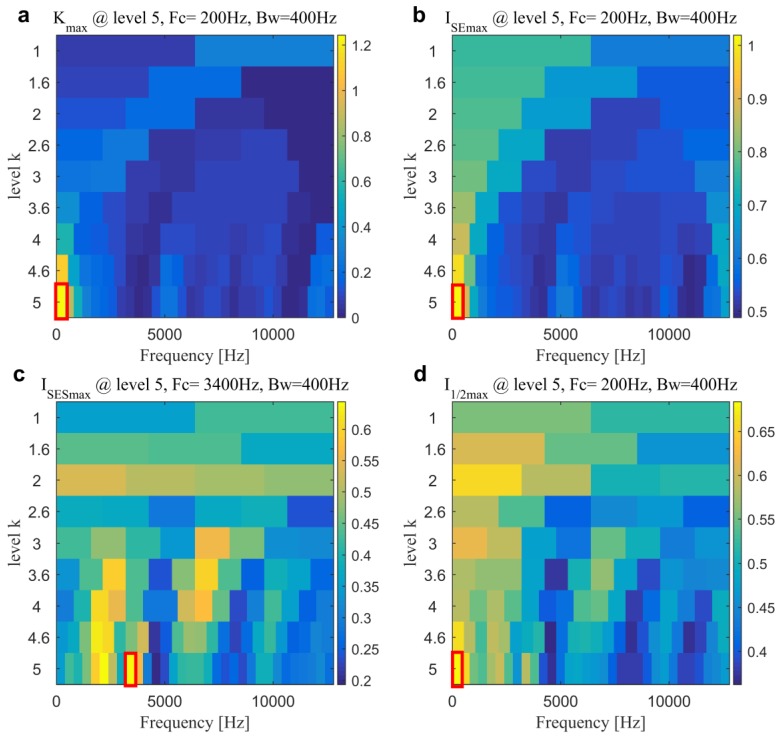
Kurtogram and infogram for the slight outer race fault diagnosis in Case 1: (**a**) kurtogram; (**b**) squared envelope (SE) infogram; (**c**) SES infogram and (**d**) average (AVE) infogram.

**Figure 8:**
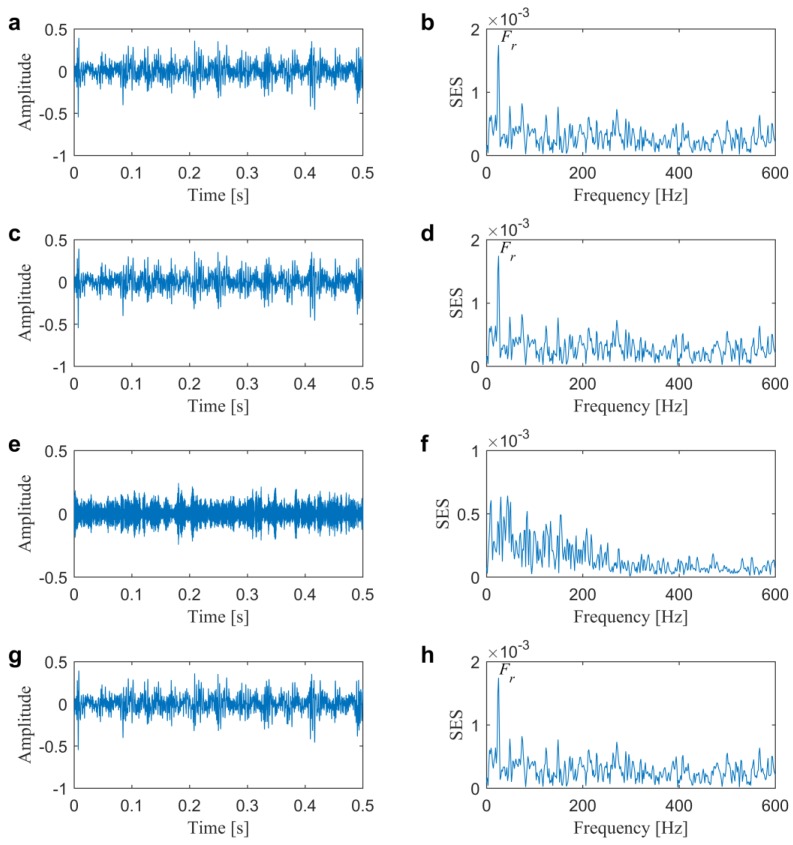
Results from kurtogram and infogram for the slight outer race fault diagnosis in Case 1: (**a**,**b**) filtered signal and obtained SES from kurtogram; (**c**,**d**) filtered signal and obtained SES from SE infogram; (**e**,**f**) filtered signal and obtained SES from SES infogram; (**g**,**h**) filtered signal and obtained SES from AVE infogram.

Subsequently, kurtogram [13] and infogram [17], as two famous fast filtering methods, are firstly conducted for comparisons to illustrate the superiority of the proposed method. Figure 7 plots four diagrams of kurtogram, SE infogram, SES infogram, and AVE infogram. We can find that kurtogram, SE infogram and AVE infogram all indicate the band with central frequency and bandwidth of 200 Hz and 400 Hz and SES infogram indicates one with central frequency and bandwidth of 3400 Hz and 400 Hz. The maximum value of the direct average negentropy is 0.6836, which is much less than the one in the previous method due to the insufficient seeking capability of infogram using fixed boundaries of the filter-bank. In addition, from the filtered signals and their corresponding SESs that are plotted in Figure 8, the results demonstrate that the selected frequency band by kurtogram, SE infogram, and AVE infogram corresponds to the low-frequency interference of rotor unbalance. It clearly evidences that the average negentropy might be easily affected by the impulsive or cyclostationary noise. Otherwise, the frequency band that is given by SES infogram is not also “informative”, because we cannot find dominate BPFO or its harmonics in Figure 8f.

**Figure 9:**
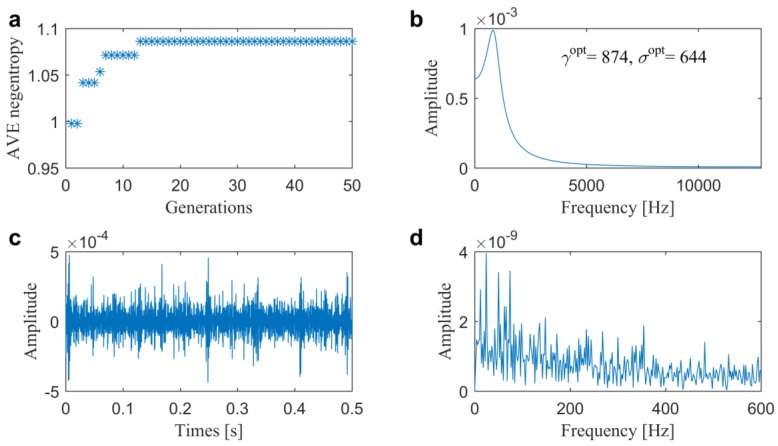
Results of the optimal filtering method 1 for the slight outer race fault diagnosis in Case 1: (**a**) fitness value; (**b**) optimal wavelet filter; (**c**) filtered signal and (**d**) its associated SES.

After that, two optimal filtering methods are also conducted for comparisons. In the first one, the AVE infogram is extended to optimal filtering in a similar way to [32], in which the parameters of ARLW are optimized by the utilized GWO using average negentropy as the single objective. From the results that are illustrated in Figure 9, we can find that the average negentropy eventually converge to 1.0860 with a center frequency of 874 Hz and bandwidth of 644 Hz in the wavelet filter that is shown in Figure 9b. Furthermore, from the filtered signal plotted in Figure 9c, we can see that the high value is from several high-amplitude impulses, which might cause higher time-domain negentropy. However, in the spectrum that is plotted in Figure 9d, there are no dominated FCFs. Therefore, it can be concluded that this method fails to detect the slight outer race fault. Thus, the direct average can hardly balance the impulsiveness and cyclostationarity in the optimization and the biggest does not mean the best, as illustrated in Figure 3, beforehand.

**Figure 10:**
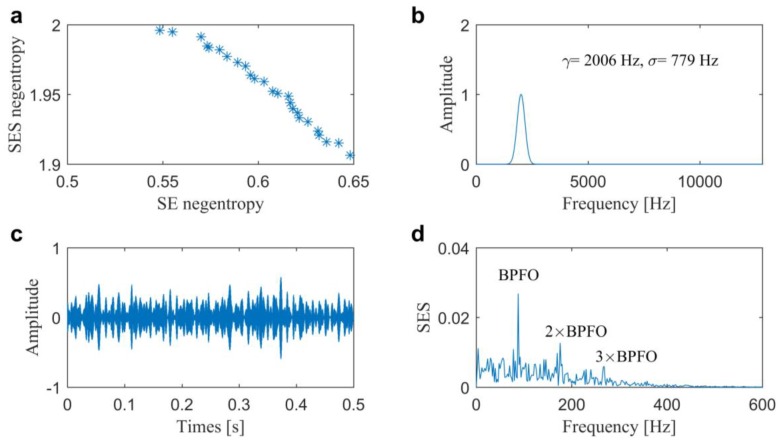
Results of the optimal filtering method 2 for the slight outer race fault diagnosis in Case 1: (**a**) Pareto front; (**b**) optimal wavelet filter; (**c**) filtered signal and (**d**) its associated SES.

In the second one, the signal plotted in Figure 6a, is further analyzed by a multi-objective optimal filtering method in our previous study [33]. With the help of Bayesian inference, the $P_{f}$ is calculated as shown in Figure 10a and the optimal complex Morlet wavelet is designed, as shown in Figure 10b with center frequency of 2006 Hz and bandwidth of 779 Hz. It is similar with the selected IFB by the proposed method due to the wavelet parameters all being multi-objective optimized by the time-domain and frequency domain negentropies. The filtered signal and its associated SES plotted in Figure 10c,d also indicate the multi-objective methods are more robust to extract the repetitive transients from the background noise.

### 3.2. Case 2: Detection of Slight Artificial Inner Race Fault in a Ball Bearing

**Figure 11:**
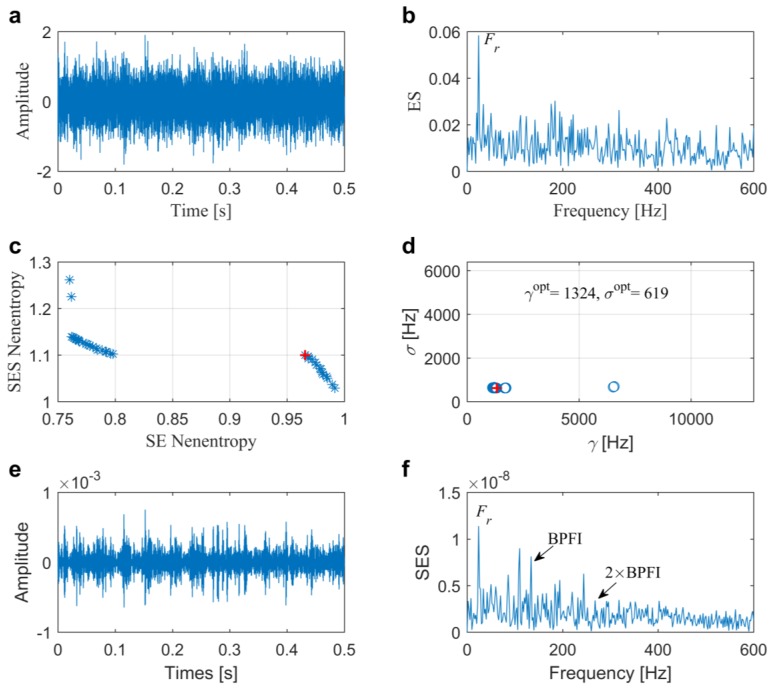
Results of the proposed method for the slight inner race fault diagnosis in Case 2: (**a**) raw signal; (**b**) envelope spectrum; (**c**) Pareto front; (**d**) distribution of the Pareto set; (**e**) filtered signal and (**f**) its associated SES.

In this case, the proposed method is further tested by an experimental signal that was collected from another bearing with an inner race fault of the same size as previous, which is subsequently installed in the set-up shown in Figure 5. Raw signal and its direct envelope spectrum from the test bearing are plotted in Figure 11a,b, respectively. Using the addressed approach, the $P_{f}$ and $P_{s}$ are presented in Figure 11c,d, with the best solution being marked by red **+**. The maximum average negentropy is calculated as 1.0329. We can clearly identify the fault-induced impulses in the temporal waveform after band-pass filtering by the optimal ARLW with center frequency of 1324 Hz and bandwidth of 619 Hz, as shown in Figure 11e. Besides, in the corresponding SES that is shown in Figure 11f, we can easily detect BPFI and the side-frequency components, which suggests that there is an inner race fault in the tested bearing.

**Figure 12:**
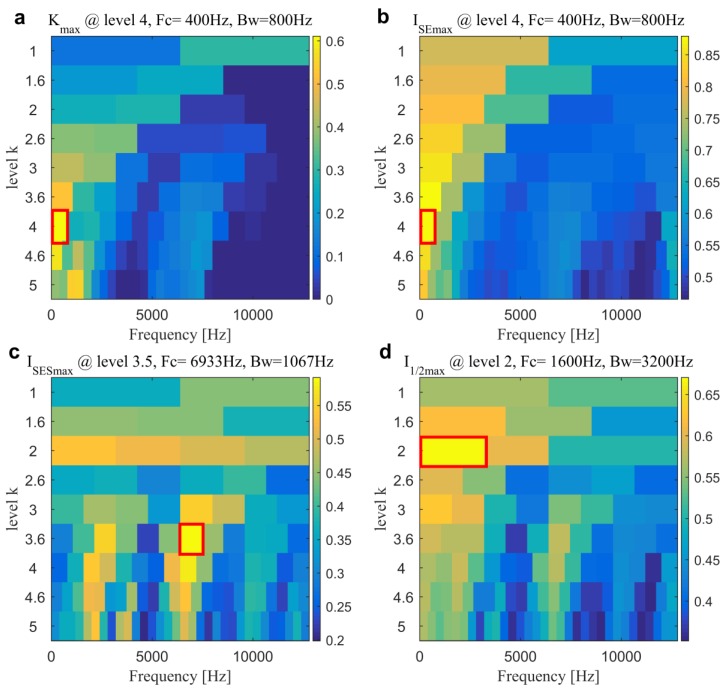
Kurtogram and infogram for the slight inner race fault diagnosis in Case 2: (**a**) kurtogram; (**b**) SE infogram; (**c**) SES infogram and (**d**) AVE infogram.

**Figure 13:**
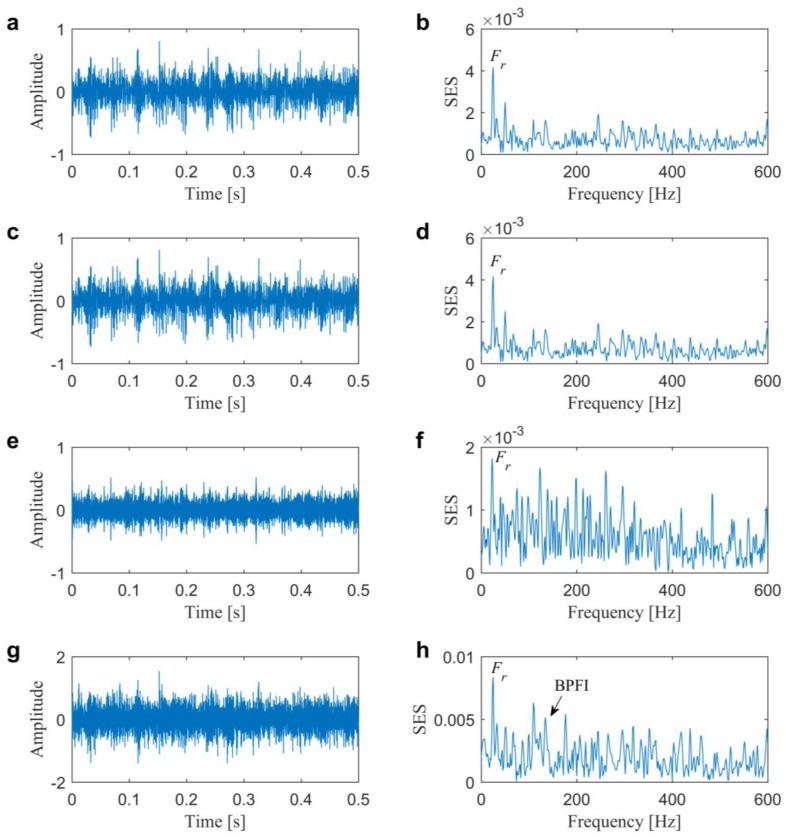
Results from kurtogram and infogram for the slight inner race fault diagnosis in Case 2: (**a**,**b**) filtered signal and obtained SES from kurtogram; (**c**,**d**) filtered signal and obtained SES from SE infogram; (**e**,**f**) filtered signal and obtained SES from SES infogram; (**g**,**h**) filtered signal and obtained SES from AVE infogram.

As comparisons, the fast filtering methods are also first conducted for the IFB selection. The kurtogram, SE infogram, SES infogram and AVE infogram are plotted in Figure 12. We can find that kurtogram and SE infogram indicate the band with central frequency and bandwidth of 400 and 800 Hz. SES infogram indicates one with central frequency and bandwidth of 6933 and 1067 Hz. After averaging the time-domain and frequency-domain negentropies, the AVE infogram selects a band with central frequency and bandwidth of 1600 Hz and 3200 Hz by the maximum average negentropy of 0.6706. From the filtered signals and their associated SESs that are plotted in Figure 13, one might find that only the last AVE infogram can detect the slight inner race fault but the time-domain waveform in Figure 13g is less impulsive compared with the result plotted in Figure 11e and the BPFI is not prominent enough in the spectrum in Figure 13h than the previous in Figure 11f. Accordingly, the fast filtering methods all need to be further improved, especially for the diagnostic of weak faults. In addition, the results also evidence that the impulsiveness as well as the cyclostationarity are all crucial in finding the IFB.

**Figure 14:**
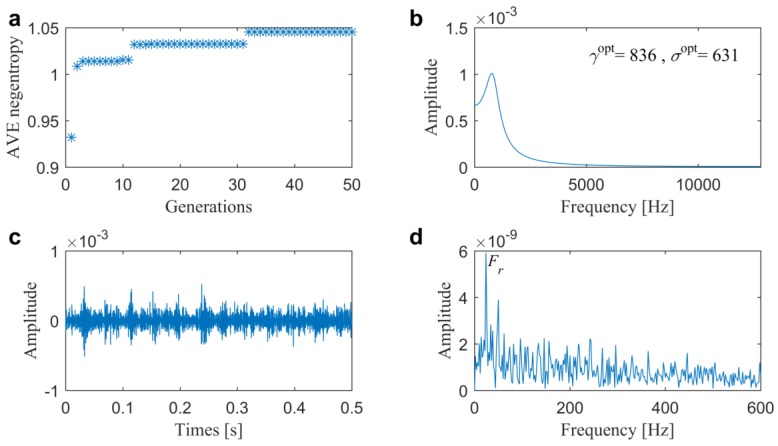
Results of the optimal filtering method 1 for the slight inner race fault diagnosis in Case 2: (**a**) fitness value; (**b**) optimal wavelet filter; (**c**) filtered signal and (**d**) its associated SES.

In the same way, two optimal filtering methods are also analyzed in this case. In the first one, Figure 14 illustrates the results from extension of AVE infogram using the single-objective GWO. We can find that the average negentropy eventually converge to 1.0450 with center frequency of 836 Hz and bandwidth of 631 Hz in the wavelet filter, as shown in Figure 14b. Furthermore, from the time-domain waveform that is plotted in Figure 14c, we can find any fault-induced impulses and in the frequency-domain spectrum plotted in Figure 14d, only the rotating frequency $F_{r}$ rather than the FCFs can be identified. The comparison results all illustrate that the method fails to detect the incipient fault once again.

**Figure 15:**
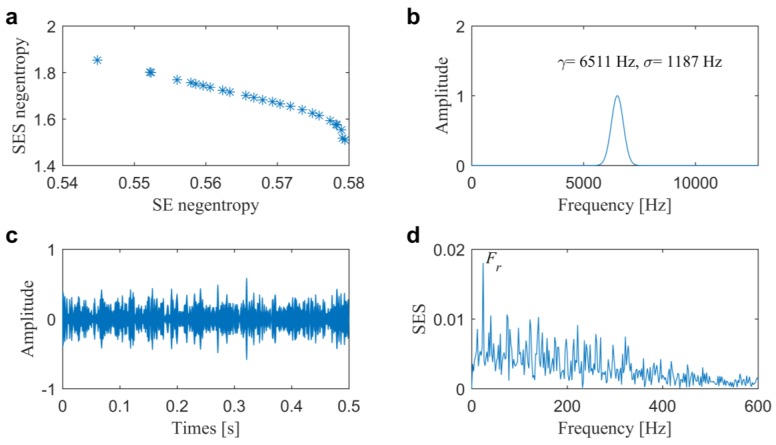
Results of the optimal filtering method 2 for the slight inner race fault diagnosis in Case 2: (**a**) Pareto front; (**b**) optimal wavelet filter; (**c**) filtered signal and (**d**) its associated SES.

In the second one, Figure 15 illustrates the results that are given by the multi-objective method while using Bayesian inference [33]. From that, we can find the optimal complex Morlet wavelet is designed with center frequency of 6511 Hz and bandwidth of 1187 Hz. Moreover, we cannot find the fault-induced repetitive transients in the filtered signal and dominant BPFI, as well as the sideband frequencies in the SES. There are two reasons for its failure in detecting the slight inner race fault. One is the complex Morlet wavelet, which is symmetric. The other is due to the weakness of Bayesian inference in global searching.

In addition, the efficiencies of the proposed as well as the compared methods are also tested in a computer with Inter^®®^ Core™ i7-4790 CPU @ 3.60 GHz and RAM of 8 GB. Table 2 lists the results, from which we can find that the proposed method has no obvious advantage in efficiency, but it has significant advantages in its effectiveness.

### 3.3. Case 3: Tracking IFB for a Condition Monitoring Data Set

**Figure 16:**
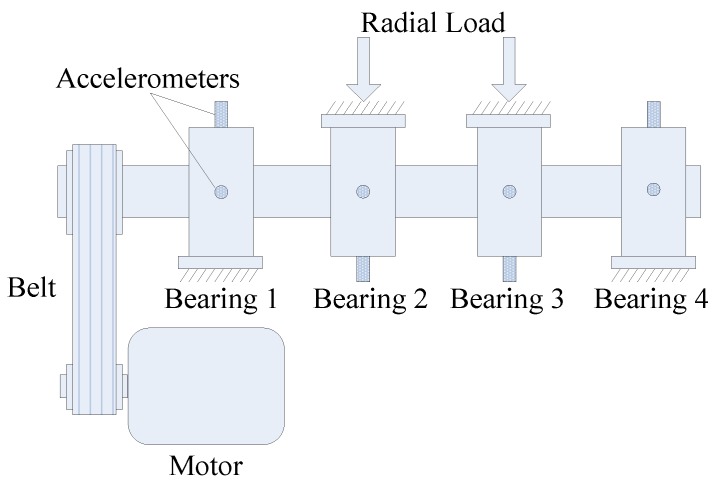
Experimental set-up for tracking the informative frequency band in Case 3.

In the above subsections, the results and comparisons in slight fault diagnosis illustrate the effectiveness of the proposed method. Furthermore, the stability of the proposed method is tested on a condition monitoring data set from [43], which is carried out for 164 h using the set-up shown in Figure 16 with an outer race defect occurred in Bearing 1 at the end. The sampling frequency is equal to 20 kHz and the length of each individual signal is equal to 1 s. The rotating speed of the shaft is set to 2000 rpm, and the fault characteristic frequency of outer race BPFO is 235.4 Hz. More details regarding this test-to-failure experiment can be found in [44].

**Figure 17:**
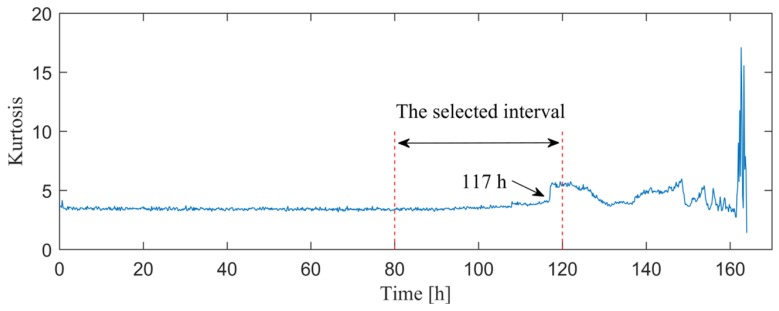
Kurtosis for the whole life cycle.

**Figure 18:**
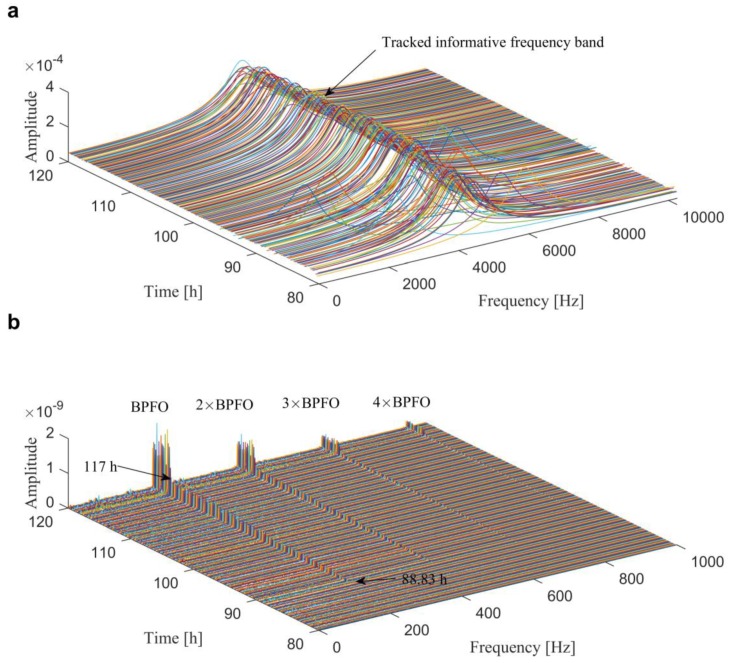
Results of the proposed method for tracking the informative frequency band in Case 3: (**a**) wavelet filters; (**b**) SESs of the filtered signals.

Before the analysis, Figure 17 presents the kurtosis of each individual in the entire life cycle, which reveals that the kurtosis started to grow after about 90 h, and jumped when the test had been carried out for 117 h. When considering the time cost, only 240 individuals in the interval of 80–120 h are analyzed. Figure 18a,b illustrate the optimal ARLW filters of each individuals obtained from the proposed method and the SESs of the associate filtered signals, respectively. From the results, we can find that the novel multi-objective method can detect the outer race fault at 88.83 h due to the dominated BPFO and its harmonics in the SES. More important is that the results also indicate that the addressed method has good stability and robustness in locating the IFB and extracting the repetitive transients during the development of fault.

## 4. Conclusions

In this paper, a novel IFB selection technique that is based on multi-objective optimized wavelet filter is reported to extract the repetitive transients in fault diagnosis of rolling element bearings. Two independent objective functions are properly selected in the optimization of ARLW parameters to combine the impulsiveness and cyclostationarity in the fault features extraction. One is to maximize negentropy of the SE and the other is to maximize that of the SES. With the help of MOGWO, some Pareto solutions can be obtained to eliminate the impulsive or cyclostationary noises. Subsequently, the optimal parameters of the filter could be selected among the non-dominated solutions that are based on the average negentropy to combine and balance the impulsive and cyclostationary features. The analysis results of two real fault signals of rolling element bearings and the comparisons with some fast and optimal filtering strategies all indicate the effectiveness of the proposed method. Moreover, the IFB tracking and fault diagnosis of a condition monitoring data set also show its stability and robustness.

## Figures and Tables

**Table 1:** Parameters of test NSK 6205RS bearing.

d (mm)	D (mm)	n	ϕ (deg)	$F_{r}$ (Hz)
7.9	39.0	9	0	24.6

**Table 2:** Time-consuming of the proposed and compared methods.

Cases	Kurgogram	SE/SES/AVE Infogram	Optimal Filtering Method 1	Optimal Filtering Method 2	Proposed Method
Case 1	0.24 s	0.14/0.19/0.31 s	1.97 s	4.89 s	4.70 s
Case 2	0.24 s	0.15/0.19/0.32 s	1.29 s	2.37 s	4.01 s
